# Determining coronavirus disease 2019 (COVID-19) community incidence threshold for preoperative testing

**DOI:** 10.1017/ice.2022.206

**Published:** 2022-08-15

**Authors:** Judy Zhou, Christopher Wituik, Mohammed Al-Salem, Alanoud Aljarbou, Jonah Dekker, Saba Karimi, Dominik Mertz

**Affiliations:** 1 Faculty of Health Sciences, McMaster University, Hamilton, Ontario, Canada; 2 St. Joseph’s Healthcare Hamilton, Hamilton, Ontario, Canada; 3 Hamilton Health Sciences, Hamilton, Ontario, Canada; 4 Department of Pediatrics, College of Medicine, Imam Mohammad Ibn Saud Islamic University, Riyadh, Saudi Arabia

## Abstract

Preprocedural testing for severe acute respiratory coronavirus virus 2 (SARS-CoV-2) is frequently used to reduce perioperative morbidity and mortality during the pandemic. Such testing is resource intensive, and the relative benefits depend on local epidemiology. We propose a threshold of 20 per 100,000 unlinked cases to activate such testing to optimize the yield and positive predictive value.

A patient may be asymptomatically, or more notably, presymptomatically infected with severe acute respiratory coronavirus virus 2 (SARS-CoV-2) at the time of a surgical procedure,^1^ potentially increasing the risk of both postprocedural mortality^2,3^ and the risk of transmission. Previous studies have reported that that the utilization of preoperative testing may be beneficial in certain settings.^4–6^

Although one can argue that all patients should be routinely tested, such testing is costly and can reduce resources that could be used more efficiently elsewhere.

Importantly, the epidemiology of coronavirus disease 2019 (COVID-19), and as such, the potential value varies by region and over time. We aimed to identify a threshold above which preprocedural testing may be justified by analyzing the data from the first 3 local waves of the COVID-19 pandemic.

## Methods

This study was conducted at 2 tertiary-care centers in Ontario, Canada. Patients who underwent elective, urgent, emergent surgical, or other major procedures and had been tested for SARS-CoV-2 with a nasopharyngeal swab within 48 hours prior to the procedure were included. Routine preprocedural testing was conducted from April to September 2020 (first wave) and from December 2020 to June 2021 (second and third waves). Study approval was provided by the Hamilton Integrated Research Ethics Board (HIREB).

A polymerase-chain reaction (PCR) assay for the SARS-CoV-2 envelope and 5’-untranslated region genes were performed. Based on cycle threshold (Ct) and availability of repeated tests, we categorized positive cases as follows. A Ct value >30 in a single test or >25 followed by negative test or test with a higher Ct value was considered remote infection. A Ct value <25 in the first positive or negative repeated test was considered an active infection. Cases with a Ct value of 25–30 and no second test were considered indeterminate. Repeated positive tests within 90 days were excluded.

Weekly unlinked cases (ie, positive cases with no travel history, no known close contact, nor outbreak exposures) were used to calculate the 7-day community incidence rate. COVID-19 is a disease reportable to local public health units, which collected this information throughout the study period. The rationale for using community instead of the total incidence in the primary analysis was that patients with known exposures would have been tested, and the procedure is routinely postponed by default. Given that this granularity in the data may not be available everywhere, we conducted a secondary analysis with the total incidence.

Firstly, the yield of preprocedural testing was stratified by the incidence rate using manual visual binning (PASW 18 software, IBM, Armonk, NY). Secondly, to summarize the ability of each incidence metric to predict active infection, receiver operator characteristic (ROC) curves were constructed with infection type (active vs remote infection, or negative test) as the dependent variable, and either community or total incidence as the independent variables (R version 3.6.3 software, R Foundation for Statistical Computing, Vienna, Austria).^7^ The Delong test was used to calculate correlated ROC curves.^8^ Cases deemed indeterminate were excluded from this analysis.

## Results

During the first wave until preprocedural testing had been paused (April 13, 2020 to September 3, 2020), 1,760 patients met the inclusion criteria (n = 948 females, 54%); their average age was 60 years (range, 0–95). The most frequent procedures included abdominal surgery (n = 437, 24.9%), endoscopy (n = 286, 16.3%), cardiac surgery or cardiac procedure (n = 270, 15.3%); urologic procedures (n = 168, 9.6%), and orthopedic procedures (n = 165, 9.4%). Only 6 tests (0.34%) returned positive, and only 1 test (0.06%) met the criteria for active infection. At the peak of the first wave (May 2020), the highest total incidence was 6.9 cases per 100,000 population and a hospitalization rate of 5.4 hospitalization per 100,000 cases. Local case numbers were underestimated because of very narrow testing criteria based on travel history and severity of symptoms in the early days of the first wave; thus, data from this first wave were not considered for the analysis of a threshold.

During the second and third waves, 10,884 total preprocedural tests were performed. Among them, 86 (0.78%) returned positive. Among positive specimens, 18 (20.9%; 0.17% of all tests) met our definition of an active infection, 59 (69.9%; 0.53% of all tests) were considered remote positives, and 9 (0.83%) had indeterminate results. The yield with weekly community incidence of <20 per 100,000 population was 2 active cases (0.05%, 9.1% of the 22 positives), which increased to 8 (0.21%, 28.6% of the 28 positives) between 20 and 35 per 100,000 population and to 8 cases (0.25%, 22.2% of the 36 positives) above 35 per 100,000 population (Table 1).

**Table 1.** Positivity Rate of Preprocedural Tests Stratified by the Weekly Community Incidence of SARS-CoV-2 Infections

Weekly Community Incidence^a^	Tests Done	Positive Cases (% of Total)	Active Infections (% of Total, % of Positive Cases)
<20	3,985	22 (0.55)	2 (0.05–9.09)
20–35	3,747	28 (0.75)	8 (0.21–28.57)
>35	3,152	36 (1.14)	8 (0.25–22.22)
Total	10,884	86 (0.70)	18 (0.17–20.9)

a Cases per 100,000 population per week.

**Fig. 1.**
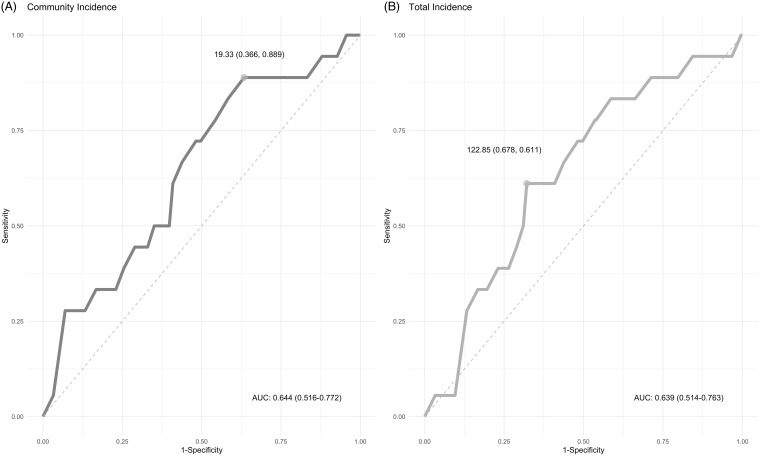
Receiver operating characteristic (ROC) curves. Active infection versus remote infection or negative as a function of (A) community incidence, and (B) as a function of the total incidence in 100,000 cases per week.

Using ROC curves, despite having limited discrimination, the best balance of sensitivity and specificity of a positive test indicating an active infection was identified at a community incidence of 19.3 per 100,000 population per week (Fig. 1). The area under the ROC curve was mathematically (but not statistically) larger when using community incidence (0.66, 95% confidence interval [CI],0.52–0.77) than when using total incidence (0.64; 95% CI, 0.51–0.76) which had a cutoff of 122.9 cases per 100,000 population per week (Delong test *P* = .7288).

## Discussion

The yield and positive predictive value of a positive SARS-CoV-2 test is a function of the pretest probability, and, as such, of the local epidemiology. As expected, the yield and positive predictive value showed a gradual increase with an increase in community transmission. Although no entirely clear threshold exists, the best possible cutoff to trigger preprocedural testing was identified as >20 of 100,000 population or unlinked cases per week, or a total incidence of >120 per 100,000 population, the latter to be used if unlinked community cases are not available in the region of interest. The use of such a threshold allowed us to reduce the unnecessary delay of procedures due to no longer active or false-positive cases^9^ while maintaining the benefit of identifying currently asymptomatic patients.

In the prevaccine era, the risk of an increase in perioperative morbidity and mortality up to 7 weeks after an infection was the main rational for such testing.^3,10^ Although this was most pronounced in those with preoperative respiratory symptoms, it seemed to also affect those in the presymptomatic stage, resulting in the recommendation for asymptomatic testing.^2^ Importantly, the extent to which this rationale still applies in a largely vaccinated population remains unclear. Therefore, the potential downsides of such testing must be considered: the resources required, the large proportion of remote infections that result in unnecessary cancellations of procedures with the associated risk of harm to patients, and the negative impact on the efficiency of the healthcare system. Another limitation when generalizing our findings to another setting is the potential difference in testing strategies. At the time of the study, all individuals with symptoms of potential COVID-19 would have qualified for testing, as would have known contacts of confirmed cases. Finally, a small proportion of patients were from outside our public health unit; however, the epidemiology was very similar in adjacent regions. There are no generally accepted Ct cutoff values to determine active infections given the variability as well as the limitations in cross-platform comparability. Therefore, we used a combination of repeated test results, when available, to determine the likelihood of active infection. Finally, the capacity to conduct contract tracing also influences the proportion of cases considered unlinked and can as such the metric of community incidence.

In conclusion, if the potential benefit of preprocedural asymptomatic testing is deemed to justify such testing, we propose that the decision should take the local epidemiology into account because the yield and, as such, the harm–benefit ratio heavily depend on the pretest probability.
